# Protective effects of silver nanoparticles in isoproterenol-induced myocardial infarction in rats

**DOI:** 10.3389/fmed.2022.867497

**Published:** 2022-08-25

**Authors:** Wawaimuli Arozal, Edwina Rogayah Monayo, Agian Jeffilano Barinda, Dian Pribadi Perkasa, Vivian Soetikno, Nafrialdi Nafrialdi, Melva Louisa

**Affiliations:** ^1^Department of Pharmacology and Therapeutics, Faculty of Medicine, Universitas Indonesia, Jakarta, Indonesia; ^2^Doctoral Program in Biomedical Sciences, Faculty of Medicine, Universitas Indonesia, Jakarta, Indonesia; ^3^Faculty of Medicine Universitas Negeri Gorontalo, Gorontalo, Indonesia; ^4^Metabolic, Cardiovascular and Aging Cluster, The Indonesian Medical Education and Research Institute, Faculty of Medicine Universitas Indonesia, Jakarta, Indonesia; ^5^Research Center for Radiation Process Technology, Research Organization for Nuclear Energy, National Research and Innovation Agency, Jakarta, Indonesia

**Keywords:** myocardial infarction, isoproterenol, silver nanopaiticles, mitochondrial dysfunction, oxidative stress, inflammation

## Abstract

**Background:**

Silver nanoparticles (AgNPs) are widely used in the medical field, including cardiovascular. However, limited research has investigated the effect of AgNPs on the protection of myocardial infarction (MI).

**Objectives:**

Isoproterenol (Iso)-induced MI and the cardiac protection offered by AgNPs were investigated in the present study. Additionally, we characterized the profile of Ag in the form of nanoparticles.

**Methods:**

Twenty-four male Wistar rats were randomly divided into four groups as follows: normal, Iso, Iso + AgNO_3_, and Iso + AgNP groups. AgNPs and silver ion (AgNO_3_) were administered intraperitoneally at 2.5 mg/kg BW for 14 days. Iso induction was performed using two doses of 85 mg/kg BW given subcutaneously on days 13 and 14. Blood and cardiac tissue samples were taken 24 h after the last dose of Iso and checked for Creatine Kinase-MB (CK-MB), lactate dehydrogenase in plasma along with oxidative stress parameters, mitochondria biogenesis markers, and inflammation representative genes in cardiac tissue. Additionally, we analyzed the histopathological features in cardiac tissue.

**Results:**

The silver was confirmed in the form of nanoparticles by its size at intervals of 8.72–37.84 nm. Both AgNO_3_ and AgNPs showed similar cardioprotective effects, as shown by the decrease in biochemical markers of cardiac toxicity, namely, CK-MB. Additionally, AgNPs group have better efficacy compared with AgNO_3_ group in ameliorating Iso-mediated oxidative stress production, as evidenced by the significant decrease in malondialdehyde level and increased superoxide dismutase activity (*P* < 0.0001 and *P* < 0.01, respectively) in cardiac tissue compared with the Iso group. Mechanistically, AgNPs, but not AgNO_3_, enhanced the expression levels of mitochondrial transcription factor A and peroxisome proliferator-activated receptor-gamma coactivator 1-alpha in post-MI heart and reduced the protein expression of nuclear factor-kappa B (NF-κB) assessed by western blot analysis. Furthermore, these results were confirmed with the histopathological evaluation of cardiac tissue. Nevertheless, pretreatment with either AgNO_3_ or AgNPs improved the aspartate aminotransferase level.

**Conclusion:**

These results suggested that AgNPs have more superior cardioprotective effect compared with AgNO_3_ against Iso-induced MI, at least in part through amelioration of NF-κB expression level induced by oxidative stress overproduction.

## Introduction

Nanotechnology is a promising technology with potential advantages in treating and preventing disease. Among the metal nanoparticles, silver nanoparticles (AgNPs) is the most widely used nanoparticle in biomedical-related products because of their broad-spectrum antimicrobial activity (1). Recently, AgNPs have been reported to have antioxidant activity (2–4) in which the antioxidant/pro-oxidant balance of AgNPs was influenced by the coating agent used for the preparation of AgNPs (4). Of note, AgNPs also showed anti-inflammatory property (3, 5, 6) by inducing M1 macrophage apoptosis and M1-to-M2 macrophage repolarization (6). Several *in vivo* studies have been reporting a promising result of AgNPs for the treatment of inflammatory disease, such as for neuropathy in diabetic rats (3), dextran sodium sulfate-induced colitis in mice (5), streptozotocin-induced hepatotoxicity in rats (7), diethyl nitrosamine-induced hepatocarcinogenesis in mice (8), alloxan-induced diabetes in mice (9), and collagen-induced rheumatoid arthritis in mice (6). Contrary to their therapeutic effects, there were some established toxicology issues in the use of AgNPs, such as cytotoxicity, mitochondrial dysfunction, autophagy, immunological response, and even cell death (10–12). Therefore, it remains challenging to find a new formulation of AgNPs with minimal side effects.

Myocardial infarction (MI) still remains the most common cardiovascular disease that contributes to the highest mortality rate in the world (13). Inflammatory response plays a pivotal role in MI progression. Of note, reactive oxygen species (ROS) production has been known to induce NF-κB-mediated MI inflammation (14, 15). However, there is no established drug that may prevent excessive ROS production and inflammation, and thus further protect MI development (16).

Isoproterenol (Iso)-induced MI is a well-known model for studying the pathophysiology of myocardial ischemia. Iso is an adrenergic agent that stimulates heart rate and contractility, thus, increasing myocardial oxygen consumption (17, 18). The excessive increase of heart stimulation may lead to ischemia and consequently causing MI. During ischemia and infarction, myocytes undergo anaerobic metabolism with less ATP production that can harm membrane integrity leading to calcium overload and myocardial dysfunction (19). Abrupt oxygen supply after ischemia insult often generates radical oxygen species, especially by mitochondria, that further damages myocytes and lead to necrosis (10–12). Furthermore, sufficient evidence supports that the expression of PGC-1α and mitochondrial transcription factor A (TFAM) were reduced in many heart failure animal models and are accompanied by oxidative stress and mitochondrial dysfunction (20). Additionally, nuclear factor-kappa B (NF-κB) is associated with NOD-like receptor superfamily, pyrin domain containing 3 (NLRP-3)inflammasome pathway that highly expressed in cardiac dysfunction (21), it will be attractive to targeting these genes for MI condition.

In the present study, we were interested in synthetizing novel AgNPs and evaluating the antioxidant and anti-inflammatory properties of AgNPs in the Iso induced MI model in rats compared with those of the conventional form of silver (AgNO_3_). Additionally, AgNPs, which probably modulate mitochondria biogenesis, will be analyzed among the treatment groups. Finally, we also performed the safety profiles of AgNPs by checking the liver and kidney functions.

## Materials and methods

### Nanoparticles

Materials used in this research were silver nitrate salt (AgNO_3_; pro analytical grade, Merck KGaA, Darmstadt Germany), sodium alginate (molecular biology grade, Himedia, India), calcium nitrate tetrahydrate (Ca(NO_3_)_2_; analytical grade, Merck, Germany), 67% (w/w) nitric acid (HNO_3_; pro analytical grade, Merck, Germany), and hydrochloric acid (HCl; pro analytical grade, Merck, Germany). All chemicals were used without further purification.

### Radiosynthesis and characterization of alginate-stabilized silver nanoparticles

Alginate-stabilized AgNPs were synthesized using gamma-ray irradiation based on the procedure used in Perkasa et al. (22) with minor modification. Briefly, the reaction system was a 30-mL aqueous solution in the sealed dark bottle, which consisted of 7.78-mM AgNO_3_ salt and 1.2% (w/v) alginate without the addition of a reducing agent or a hydroxyl radical scavenger. Radiation processing was conducted via gamma-ray irradiation at a dose of 20 kGy using Gammacell 220 cobalt-60 irradiator facility (series Gammacell 220, MDS Nordion, Canada). Successful conversion of silver ion into AgNPs was detected based on colorimetric analysis by using Ultraviolet–visible (UV–Vis) spectrophotometer (series Cary100, Agilent, United States) through an absorption band of localized surface plasmon resonance (lSPR) peaked at approximately 400 nm. Then, the colloidal AgNPs were stored at 4°C under dark conditions until used for further experiment.

The AgNP concentration was measured on the basis of the procedure conducted by Dong et al.(23) with modification. Briefly, the sample for total silver measurement was prepared by acidifying 1 mL of colloidal AgNPs with 9 mL 67% (w/w) HNO_3_ overnight at 37°C. Then, the digested suspension was diluted with ultrapure water to a final HNO_3_ concentration of 0.2% (w/v). The sample for silver ion measurement was prepared by pretreating 5 mL of each colloidal AgNP with 5 mL of 4% (w/v) calcium nitrate solution followed by centrifugation at 1,000 × *g* for 10 min. Then, the supernatant was carefully collected for silver ion analysis. Total silver and silver ion concentrations were measured using a flame atomic absorption spectrometer (series 240FS AA, Agilent, United States).

The AgNP concentration was calculated using **Eq.** (1):


(1) [A⁢g⁢N⁢P⁢s]=[t⁢o⁢t⁢a⁢l⁢s⁢i⁢l⁢v⁢e⁢r]-[s⁢i⁢l⁢v⁢e⁢r⁢i⁢o⁢n]

where [*AgNPs*], [*total silver*], and [*silver ion*] are the concentrations of AgNPs, total silver, and silver ion in mg/mL, respectively. The morphology of nanoparticles was observed using a transmission electron microscope (TEM; series JEM-1400, JEOL, Japan), whereas particle size distribution was analyzed on the basis of electfon microscope image using ImageJ (ver. 1.51i, developed by Wayne Rasband, National Institute of Health, United States) software. The hydrodynamic diameter and zeta potential of nanoparticles were measured using dynamic light scattering (DLS; series Zetasizer Nano, Malvern Panalytical Ltd., United Kingdom).

### Myocardial infarction *in vivo* model

The Health Research Ethics Committee of the Faculty of Medicine, Universitas Indonesia (Ethics No.KET-1309/UN2.F1/ETIK/PPM.00.02/2020) approved this study. The male Wistar rats (weight 190–220 g) were obtained from an animal research breeding facility (BPOM Laboratory, Jakarta, Indonesia); housed at a temperature of 22 ± 3°C, humidity of 55%, and a 12-h/12-h light/dark cycle; and fed with standard pellet and water *ad libitum*. Twenty-four rats were divided randomly into four treatment groups: normal, Iso, Iso + Ag (Iso + AgNO_3_ 2.5 mg/kg BW), and Iso + AgNPs (Iso + AgNPs 2.5 mg/kg BW) groups. Iso (purchased from Sigma-Aldrich) was dissolved in sodium chloride 0.9% and injected subcutaneously at a dose of 85 mg/kg BW at days 13 and 14 of the study. The dose of Iso was selected on the basis of a previous research (24).

**FIGURE 1:**
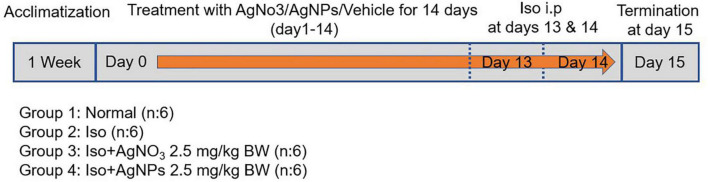
The timeline of *in vivo* study.

Radio-synthesized AgNPs were used directly for animal experimentation without further purification. Residual silver ions were passivized by adding 7.78-mL sodium chloride salt. Simultaneously, 0.41-mg/mL silver ions were prepared by dissolving 20.61-mg AgNO_3_ salt in 30 mL of ultrapure water. Test materials were kept at 4°C under dark conditions along the animal experimentation period. AgNO_3_ and AgNP were injected intraperitoneally (IP) at 2.5 mg/kg BW from day 0 to day 14, as described in Figure 1 The doses of AgNO_3_ and AgNPs were selected on the basis of our preliminary study. IP method was selected because of the better delivery system particularly for penetrating macromolecule alginate that we used in inflamed cardiac tissue (25). Rats in the normal and Iso groups were injected IP with distilled water as a vehicle of AgNO_3_ and AgNPs. On day 15, all rats were sacrificed, and blood was collected for further biochemical parameter analysis. The heart was removed immediately, and tissues were washed with cold phosphate-buffered saline. A part of heart tissue was kept in formalin buffer for histopathological analysis. The remaining tissue was kept at −80°C for further molecular analysis.

### Determination of biochemical parameters

Blood samples were centrifuged (3,000 × *g*) for 15 min at 4°C. The serum was harvested and checked for kidney function parameters (urea and creatinine levels) and liver functions (alanine aminotransferase (ALT) and aspartate aminotransferase (AST) activities) using commercial kits (DiaSys, Indonesia). Additionally, we checked the cardiac toxicity parameters such as creatine kinase-MB (CK-MB) and lactate dehydrogenase (LDH) activities in serum using commercial kits (DiaSys, Indonesia).

### Histopathological evaluation

Left Ventricle cardiac tissues were fixed in 10% formalin, routinely processed, and embedded in paraffin wax. The paraffin section (5 μm) was cut on glass slides and stained with hematoxylin and eosin (H&E). A blinded pathologist examined the H&E-stained heart samples particularly in the longitudinal section. The samples were evaluated using a Leica DM 750 light microscope with 100 × magnification (Leica Microsystems Inc., Buffalo Heights, IL, United States). The myocardial pathologies were assessed according to the Association for European Cardiovascular Pathology guidelines (26).

### Determination of oxidative stress-related parameters

Cardiac malondialdehyde (MDA) levels were estimated as a marker of lipid peroxidation using the method of Okhawa et al. (27), whereas antioxidant enzymes were measured by evaluating the levels of superoxide dismutase (SOD) using commercial kits (Dogen Bio, South Korea). Protein levels were estimated using the Bradford (Coomassie Plus) assay method, using bovine serum albumin as a standard (28). Additionally, to check the oxidative stress in the circulation, we determined the total antioxidant capacity (TAOC) level in serum in spectrophotometric analysis by using a commercial kit from Sigma (MAK187) according to the manufacturer’s instructions.

### Quantitative real time polymerase chain reaction

RNA was isolated from 20 mg of cardiac tissue using the Quick-RNA MiniPrepPlus kit (Zymo Research, CA, United States) and then synthesized to cDNA using the ReverTra Ace^®^ qPCR RT Master Mix (Toyobo BioTech, Osaka, Japan) following the manufacturer’s protocols. The mRNA expressions of peroxisome proliferator-activated receptor-gamma coactivator 1-alpha (PGC-1α) and TFAM were analyzed using qRT-PCR with β-actin as a reference gene. The primer sequences used for PGC-1α and TFAM are described in Table 1. The level of mRNA expressions was calculated using the Livak method (29).

**TABLE 1:** Rat PGC-1α and TFAM PCR primer sequences.

Gene	Sequence	GenBank
PGC-1α	F: ATGTGTCGCCTTCTTGCTCT R: ATCTACTGCCTGGGGACCTT	NM_031347.1
TFAM	F: GCTAAACACCCAGATGCAAAA R: CGAGGTCTTTTTGGTTTTCC	NM_031326.1

PGC-1α, peroxisome proliferator-activated receptor-gamma coactivator 1-alphaα; TFAM, mitochondrial transcription factor A.

### Immunoblotting

Protein was extracted from myocardial tissue using Radioimmunoprecipitation assay (RIPA) buffer enriched with protease and phosphatase inhibitors (Sigma P0044; Sigma P8340). The protein concentration was equalized using Bradford (Coomassie Plus) commercial assay kit (Merck B6916) in spectrophotometric analysis at a wavelength of 595 nm (28). The isolated protein was then stored at −80°C until further analysis. The protein samples was separated with SDS-PAGE based on their molecular weights. Afterward, protein samples were transferred to a nitrocellulose membrane. The membranes were incubated with 5% skim milk in TBS-T for 30 min and labeled with the primary antibody diluted in blocking buffer overnight at 4°C, followed by treatment with secondary antibody diluted in blocking buffer. The signals were then detected using Chemiluminescence Alliance 4.7 (Uvitec) with enhanced chemiluminescence substrate (Bio-Rad). Finally, the band of the specific target proteins was visualized using gel documentation, quantified, and normalized with G Glyceraldehyde 3-phosphate dehydrogenase (GAPDH), as housekeeping gene, expression levels with the data presented in arbitrary units. The immunoblotting image densitometry was analyzed using ImageJ software as previously explained (30). For the final densitometry results, 16-bit immunoblot images in Tagged Image File format were plotted with a rectangle lane, the background intensity was adjusted, and the plotted image was calculated. We analyzed four heart tissue samples from four different animals. All antibodies for western blot analysis were purchased from Cell Signaling Technology (Beverly, MA, United States): GAPDH (14C10) Rabbit mAb (CST#2118), NF-κB p65 (D14E12) rabbit mAb (CST#8242), NLRP3 (D2P5E) rabbit mAb (CST #13158), and antirabbit IgG HRP-linked Antibody (CST #7074S).

### Statistical analysis

Data were presented as the standard error of the mean. The differences between groups were analyzed using one-way Analysis of Variance (ANOVA), with a significance limit (α) of 0.05. The analysis was continued using the least significant difference test (Tukey method). All statistical analyses were performed with SPSS version 26. All of the plots were presented in GraphPad Prism version 9.0.0.

## Results

### Characteristics of silver nanoparticles

**FIGURE 2:**
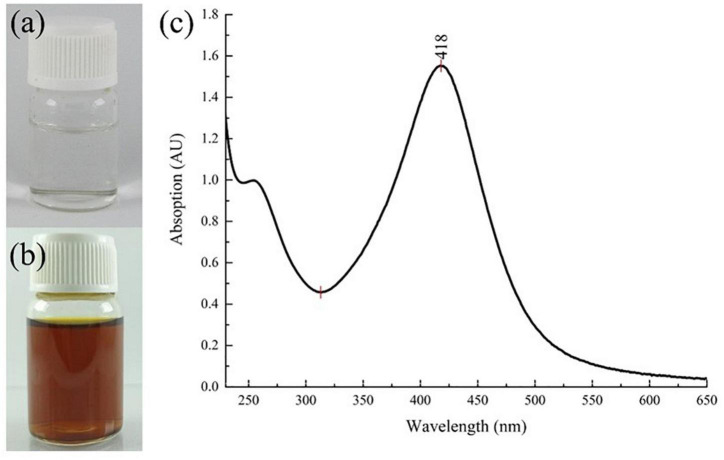
Color change of the reaction system **(a)** before and **(b)** after gamma irradiation and **(c)** the UV–Vis spectrum of colloidal AgNPs. AgNPs, silver nanoparticles.

**FIGURE 3:**
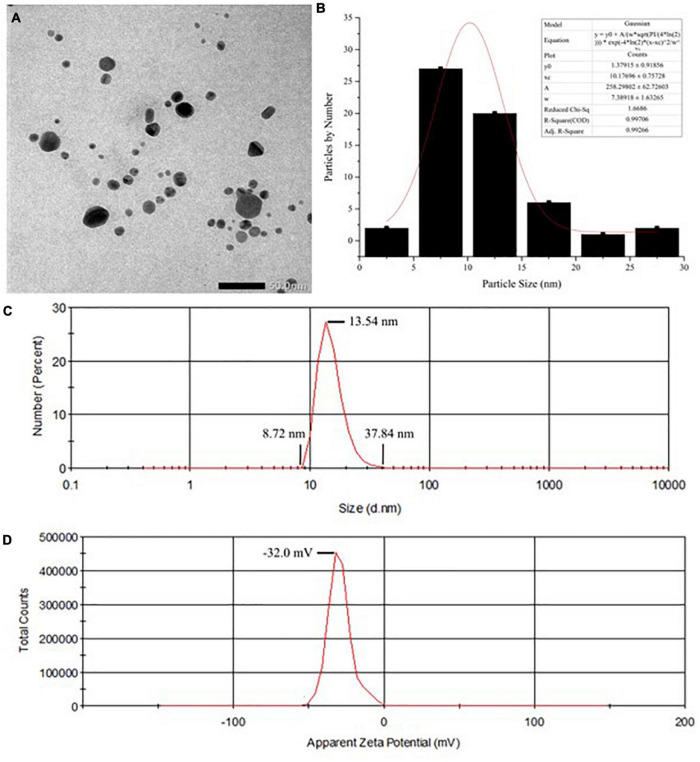
**(A)** The TEM imaging and **(B)** particle size distribution of the AgNPs. **(C)** Hydrodynamic diameters and **(D)** zeta potential of colloidal AgNPs. TEM, transmission electron microscopy; AgNPs, silver nanoparticles.

Results showed that the color of the reaction system changed from a transparent colorless suspension to a transparent yellowish–brown suspension after gamma-ray irradiation, as seen in Figures 2a,b). The color change was due to the excitation of lSPR at the visible spectrum. Accordingly, the UV–Vis spectra of the reaction system in Figure 2c showed a strong, intense peak at approximately 420 nm, which is characteristic of AgNPs (22, 31, 32). Flame atomic absorption spectrometry was used to determine the concentration of AgNPs at approximately 0.481 mg/mL. TEM imaging showed that alginate-stabilized AgNPs had a spherical shape with smooth edges, as shown in Figure 3A. The particles were well distributed and separated within the observation field. Processing on the TEM micrograph revealed that the AgNPs had a diameter size of 10.18 ± 3.69 nm (adjective R-square = 0.99), as shown in Figure 3B. However, it can be observed that there were some big particles, but their size was not more than 50 nm. The behavior of nanoparticles in suspension was determined by DLS measurement, as shown in Figures 3C,D. The hydrodynamic size of most nanoparticles was at range of 8.72 – 37.84 nm, which peaked at 13.54 nm. The hydrodynamic size of AgNPs was higher than the TEM results because of the dielectric layer at the particle and solution interface, whereas the zeta potentials of AgNPs were measured at −32.0 mV, indicating the stability of colloidal nanoparticles against spontaneous agglomeration.

### The effects of silver nanoparticles on cardiac toxicity parameters in iso-induced myocardial infarction rats

**FIGURE 4:**
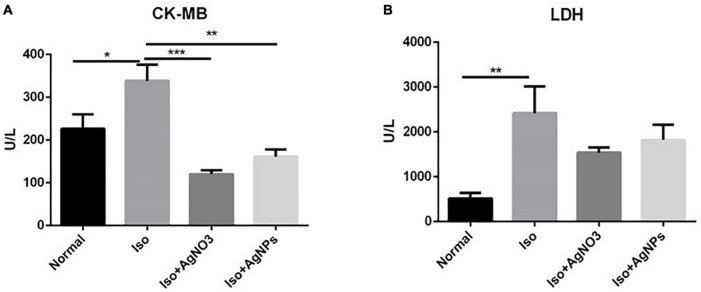
The effects of AgNO_3_ and AgNPs on CK-MB **(A)** and LDH **(B)** on Iso-induced MI in rats. Each column represents the mean ± SEM of six rats. Data analysis was performed using one-way ANOVA, followed by Tukey multiple comparison test. ***P < 0.001; **P < 0.01; *P < 0.05. CK-MB, creatinine phosphokinase isoenzyme; LDH, lactate dehydrogenase; Iso, isoproterenol; AgNO_3_, silver ion; AgNPs, silver nanoparticles.

Figure 4 shows the significant elevation of CK-MB (P < 0.05) and LDH (P < 0.01) activities in rats treated with Iso only (Iso) compared with those in normal rats. Pretreatment with AgNO_3_ (P < 0.001). and AgNPs (P < 0.01) significantly restored CK-MB but not LDH activities.

### The effects of silver nanoparticles on liver and kidney functions in iso-induced myocardial infarction rats

To investigate whether 14 days of treatment with Iso, Iso + AgNPs, or Iso + AgNO_3_ caused liver and kidney dysfunction, we next examined the serum levels of urea and creatinine and the activities of ALT and AST. Table 2 shows the significant (P < 0.01) elevation of ALT, AST, urea, and creatinine levels in the Iso group compared with those in the normal group. Pretreatment with AgNO_3_ and AgNPs decreased the AST level significantly (P < 0.05).

**TABLE 2:** Liver and kidney functions among the groups.

	Normal	Iso	Iso + AgNO_3_	Iso + AgNPs
ALT (U/L)	25.61 ± 0.8	78.45 ± 15.18^a)^	44.75 ± 5.16	56.61 ± 3.25
AST (U/L)	20.22 ± 2.17	44.57 ± 6.3^a)^	28.04 ± 2.71^b)^	27.68 ± 1.3^b)^
Plasma urea (mg/dL)	53.33 ± 2.43	119.17 ± 68.33^a)^	60.83 ± 3.86	55.00 ± 3.58
Plasma creatinine (mg/dL)	0.4 ± 0.06	1.4 ± 0.96^a)^	0.35 ± 0.06	0.35 ± 0.06

Values are given as mean ± SEM.

^*a*)^ *p* < 0.01 versus normal based on two-tailed *t*-test.

^*b*)^ *p* < 0.05 versus Iso based on two-tailed *t*-test.

### Histopathological evaluation

**FIGURE 5:**
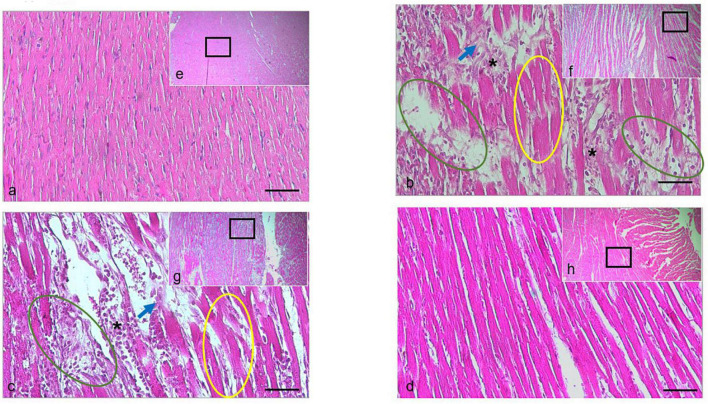
H&E staining of the cardiac tissue isolated from the rat in the normal group, or treated with Iso ± AGNO_3_ or AgNPs. **(a-d)** were presented as 200 × magnification and representative images from **(e–g)** (black rectangle) were 40 × magnification. Scale bar = 100 μm. Yellow circle shows myofibril waviness, green circle shows interstitial edema, asterisk indicates eosinophil infiltration, and blue arrow points to necrotic cardiomyocytes. The **(a,e)** normal, **(b,f)** Iso-treated, **(c,g)** Iso + AgNO_3_-treated, and **(d,h)** Iso + AgNPs-treated rat groups.

The histopathological evaluation of H&E-stained longitudinal sections of left ventricle myocardial tissue (Figure 5) from the Iso group showed the massive pathological features represented by the wavy appearance in myocardial fibers, interstitial edema, heavy eosinophil infiltration and cardiomyocytes necrotic. These data indicated the Iso was indeed-induced damage in heart tissue. Similarly, those findings can still be found in rats cotreated with AgNO_3_, but not in those cotreated with AgNPs, indicating the histological protection features in rats treated with AgNPs.

### The effects of silver nanoparticles on oxidative stress-related parameters in iso-induced myocardial infarction rats

**FIGURE 6:**
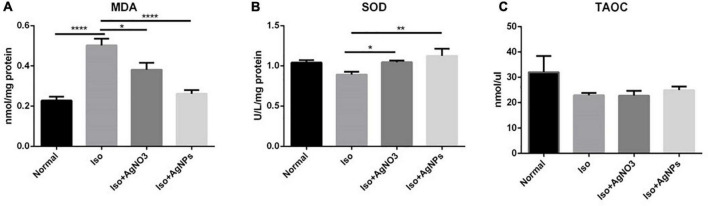
The effects of AgNO_3_ and AgNPs on the level of MDA in cardiac tissue **(A)**, SOD activity in cardiac tissue **(B)**, and the TAOC level **(C)** in serum. Each column represents the mean ± SEM of six rats. Data analysis was performed using one-way ANOVA, followed by Tukey multiple comparison test. ****P < 0.0001; **P < 0.01; *P < 0.05. Iso, isoproterenol; AgNO_3_, silver ion; AgNPs, silver nanoparticles.

To determine whether the antioxidant mechanism plays a pivotal role in the cardioprotection action of AgNO_3_ and AgNPs against MI in rats, TAOC level from circulating serum, MDA level, and SOD activity in cardiac tissue were also analyzed (Figure 6). The content of MDA were increased in the rats treated with Iso only. Conversely, in rats treated with AgNO_3_ and AgNPs, the MDA level decreases significantly (*P* < 0.05 and *P* < 0.0001) compared with rats in the Iso group. Additionally, SOD activity was increased significantly with AgNO_3_ (*P* < 0.05) and AgNPs (*P* < 0.01) pretreatment. However, no changes were found on the serum TAOC level among the groups.

### The effects of silver nanoparticles on transcription factor A and PGC-1α mRNA expression levels in iso-induced myocardial infarction rats

**FIGURE 7:**
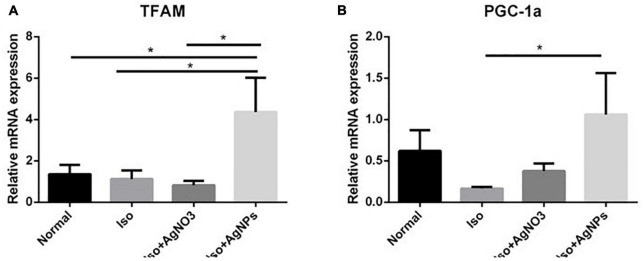
The effects of AgNO_3_ and AgNPs on the mRNA expressions of TFAM **(A)** and PGC-1α **(B)**. Each column represents the mean relative mRNA expression ± SEM of six rats. Data analysis was performed using one-way ANOVA, followed by Tukey multiple comparison test. *P < 0.05. Iso, isoproterenol; AgNO_3_, silver ion; AgNPs, silver nanoparticles.

Transcription factor A and PGC-1α mRNA expression levels were measured as markers of mitochondrial biogenesis. As we expected, we found that both TFAM and PGC-1α were significantly enhanced in rats treated with AgNPs (*P* < 0.05) (Figure 7).

### The effects of silver nanoparticles on protein expressions of NF-κB and NLRP3 in iso-induced myocardial infarction rats

**FIGURE 8:**
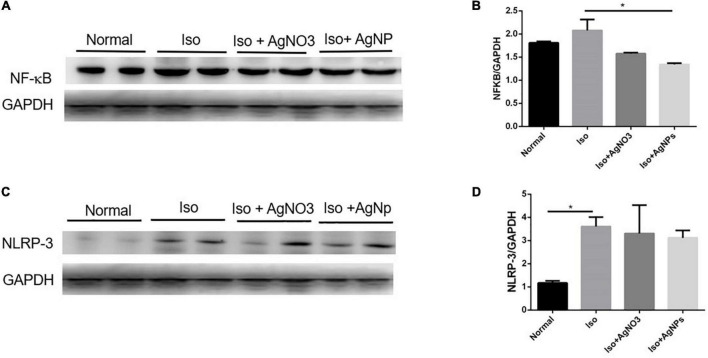
Representative western blot gel in the cardiac tissue of GAPDH, NF-κB, and NLRP3 **(A,C)**; quantified band analysis of NF-κB and NLRP3 protein expression **(B,D)**. The quantified result was normalized to GAPDH expression. Data analysis was performed using one-way ANOVA, followed by Tukey multiple comparison test. *P < 0.05. Iso, isoproterenol; AgNO_3_, silver ion; AgNPs, silver nanoparticles.

To confirm whether the inflammation was involved in Iso-induced MI in this study, we checked the protein expression of NF-κB and NLRP3 in cardiac tissue. As shown in Figures 8A,B, NF-κB protein expression level was decreased in the AgNP group than in the Iso group (*P* < 0.05). Likewise, the heart injected with Iso was significantly induced the NLRP3 inflammasome gene compared with normal rats (*P* < 0.05). However, cotreatment with either AgNO_3_ or AgNPs failed to decrease the up-regulation of the NLRP3 inflammasome gene caused by Iso (Figures 8C,D).

## Discussion

In this study, we discovered a new formulation of AgNPs (stabilized by irradiated alginate) and revealed for the first time the protective effects of AgNPs in the MI animal model. We showed that AgNPs was able to prevent Iso-induced MI by alleviating the NF-κB expression level, which induced by ROS overproduction.

This study has been successfully radiosynthesized alginate that stabilized AgNPs by using gamma ray irradiation. The nanosytem was a yellowish–brown transparent suspension. It exhibited an intense UV–Vis absorption peak in a wavelength range of 300–600 nm. Particle morphology was circular with smooth edges, which was preferred because of our silver nanoparticles was less cytotoxicity compared with that of sharp geometry nanoparticles (33, 34). The particle size was measured at 10.18 nm using TEM, and the hydrodynamic diameter was slightly higher at 13.54 nm because of the dielectric layer. The irradiated alginate, as a stabilizing agent, provided a negative surface charge with sufficient interparticle repulsion (zeta potential = −32.0 mV) against spontaneous agglomeration. Previously, alginate-stabilized AgNPs have been synthesized using plasma-activated water (35) and glucose reduction methods (36), but at larger particle sizes of approximately 22 and 70 nm, respectively. However, the glucose reduction method showed better suspension stability with zeta potential at -47 mV at alginate concentration of 0.5% (w/v) than the other nanoparticles (36).

Sufficient evidence in the cell culture and animal model studies has demonstrated that Iso, a nonselective β-adrenoceptor agonist, can cause cardiac overstimulation, leading to ischemia, severe oxidative stress, and inflammation, which can lead to MI. It can also generate free radicals and stimulate lipid peroxidation, which might be a causative factor for irreversible damage of the myocardial membrane (37, 38).

In this study, we demonstrated that the administration of Iso in rats was associated with myocardial injury as documented by a significant increase of CK-MB and LDH activities in the Iso group, an indicator of leakage of cardiac enzymes. CK-MB activity was significantly decreased in both AgNO_3_ and AgNP groups compared with the Iso group, indicating protective effects of silver on myocardial damage. Although both AgNO_3_ and AgNPs improved cardiac enzyme markers, only AgNPs could restore histological changes in the myocardium caused by Iso (Figure 5).

Mechanisms of Iso-induced myocardial damage may be multifaceted. The present study tried to investigate these possible mechanisms through three main approaches, namely oxidative stress, inflammation, and possible mitochondrial dysregulation. Therefore, possible mechanisms of protection exerted by silver in two formulations (AgNO_3_ and AgNPs) have also been elucidated.

We next evaluated the antioxidant effects of AgNO_3_ and AgNPs in Iso-treated rats. Previous studies showed that Iso-induced MI in rats caused increased MDA and decreased SOD (39). MDA is one of the oxidative stress markers produced as a by-product of polyunsaturated fatty acid peroxidation and arachidonic acid metabolism (40). The low oxygen level and oxidative stress brought on by acute ischemia damage may cause a rise in serum MDA levels in individuals with acute myocardial injury (41). In this study, our data clearly showed a significant increase of MDA level after Iso administration. The role of AgNPs in protecting against myocardial damage due to oxidative stress is observed on MDA and SOD parameters. Cotreatment with AgNPs, but not AgNO_3_, displayed a significant decrease of MDA and an increase of SOD. The mechanism by which AgNPs have a better profile than AgNO_3_ to reduce the oxidative stress caused by Iso need to be determined in a future study, particularly the content of silver in cardiac tissue. Several studies showed the pro-oxidant effects in AgNPs (42–46). Interestingly, this study found that our AgNP formulation salvaged the ROS production-mediated myocardial damage induced by Iso administration (4, 46, 47). This mechanism is obviously due to the stabilizing agent that we used for AgNP preparation. Similarly, previous studies showed the antioxidant effects of AgNP with ethylene glycol (4) and various herb extracts (48–51) as the stabilizing agents. Thereby, stabilizing agents could be an essential factor in AgNP toxicity.

Exploration into mitochondria dysregulation caused by Iso was also investigated in our study. Our data showed that 14 days of pretreatment with AgNPs, but not AgNO_3_, has dramatically enhanced the mRNA expression level of TFAM and PGC-1α, even though those mRNAs expression level in Iso group was comparable with normal group. TFAM is a transcription factor synthesized in the nucleus and transported into mitochondria. It works by stabilizing mtDNA and has beneficial effects of mitigating calcium mishandling in the myocardium caused by Iso (52). The different effects exerted by AgNO_3_ and AgNPs suggested that silver in the form of nanoparticles might solely enter the mitochondria or nucleus and induce the expression of TFAM and PGC-1α, leading to protective effects on myocardial cells. It is of interest that many published articles stated that exposure of cells to AgNPs can cause mitochondrial damage such as mitochondrial swelling, disrupting mitochondrial membrane potentials and leading to mitochondrial pathway-induced apoptosis (53) but we found the opposite effects in our study. We speculate that the green synthesized method of our AgNPs formulation by using alginate may contribute to its beneficial effects (54). TFAM and PGC-1α were analyzed in transcription level, but not in protein level. Therefore, further investigations are needed to check those genes in protein level or performed the mitochondrial function assay such as mitochondrial membrane potential assessment, mito-fussion/fission or mitochondrial oxidative stress analyses. The lack of performing those analysis was considered as a limitation of our study.

It is already established that oxidative stress may induce activation of NF-κB, a major transcription factor activated upon response to oxidative stress, and this signaling pathway plays an essential role in cell proliferation and differentiation, thus contributing to cardioprotective effects. Apart from the cardioprotective effect of AgNPs, NF-κB is also implicated as early inflammatory responses, and can induce the activity of several inflammatory cytokines such as Tumor necrosis factor alpha(TNFα), Interleukin-1 (IL-1), and Interleukin-6 (IL-6). Moreover, NF-κB appears to be detrimental to myocytes (55). Previous studies have shown the primary role of NF-κB activation in doxorubicin-induced cardiotoxicity (56, 57). In the present study, Iso administration was associated with an increase of NF-κB protein expression. Interestingly, cotreatment with AgNP has decreased the expression of NF-κB, suggesting that AgNP formulation has attenuating effects on NF-κB-derived inflammatory response. Similarly, a recent *in vitro* study using macroalgae bio-capped AgNPs reported an anti-inflammatory property (58).

NLRP3 inflammasome has been shown to contribute to myocardial dysfunction through stimulation of IL-1B and IL-18 production, and reducing its activity has been implicated in myocardial protection (59). In our study, Iso administration was shown to induce a remarkable increase of NLRP3 expression in cardiac tissue from Iso group compared with normal group. However, pretreatment with AgNO_3_ and AgNPs was unlikely to show normalization of NLRP3, suggesting that this NLRP3 pathway may not contribute to protective effects of AgNO_3_ and AgNP on Iso-induced myocardial damage.

A previous study reported that silver, whether in conventional or nanoparticle form, negatively influences several organs, including the kidney and liver (60). In the present study, 14 days of intraperitoneal administration of either AgNO_3_ or AgNPs did not show any liver and kidney dysfunction as evidenced by no significant changes of urea, creatinine, ALT, and AST activities among the groups. These data may suggest the safety profile of silver in two formulations used in our study.

## Conclusion

This study concluded that silver, specifically in nanoparticle form (AgNPs), has a cardioprotective effect against Iso-induced MI. The mechanisms of this protection are mediated, at least in part, through the amelioration of NF-κB expression level induced by oxidative stress overproduction.

## Data availability statement

The original contributions presented in this study are included in the article/supplementary material, further inquiries can be directed to the corresponding author/s.

## Ethics statement

The animal study was reviewed and approved by Ethics Committee Faculty of Medicine Universitas Indonesia.

## Author contributions

WA, EM, VS, AJB, NN, ML, and DPP designed the experiments, analyzed the data, and wrote the manuscript. WA, EM, DPP, and AJB performed the experiments and analyzed the data. All authors contributed to the article and approved the final submitted version.
